# Predicting Age-Related Hearing Loss in Community-Dwelling Older Adults: Multicenter Retrospective Cohort Study

**DOI:** 10.2196/81135

**Published:** 2025-12-10

**Authors:** Jing Li, Shuai Jin, Liu Sun, Jun-E Liu, Qiang Shen, Miao Shang, Hanting Wang, Yuanyuan Zhao

**Affiliations:** 1 School of Nursing Capital Medical University Beijing, Beijing China; 2 Dongcheng District Community Health Service Management Center Beijing China; 3 Department of Otolaryngology Beijing Tongren Hospital Beijing China

**Keywords:** primary health care, age-related hearing loss, prediction model, multicenter study, nomogram

## Abstract

**Background:**

Age-related hearing loss (ARHL) is associated with severe negative outcomes, including social isolation, depression, and cognitive decline. Despite this, routine ARHL screening is often neglected in primary care due to low awareness, resource limitations, and inefficiencies. A practical risk assessment tool could effectively address this gap.

**Objective:**

This study aims to develop and validate a user-friendly nomogram for identifying older adults at high risk of ARHL in community settings, thereby facilitating targeted screening and timely interventions.

**Methods:**

This multicenter retrospective cohort study included 34,983 older adults from 3 primary health care centers in Beijing (January 2020 to October 2023). Data from center A (n=18,707) were used for model development, with external validation performed on cohorts from center B (n=11,008) and center C (n=5268). Least absolute shrinkage and selection operator and logistic regression identified the final predictors. Model performance was evaluated using discrimination, calibration, and decision curve analysis, leading to the development of an online nomogram.

**Results:**

In the training cohort (center A), 1177 participants (6.3%) had hearing loss. Six key predictors were identified: age, education, exercise frequency, physical function, dietary habits, and hypertension. The multivariate logistic regression model demonstrated good discrimination in internal validation (area under the curve [AUC] 0.806, 95% CI 0.782-0.831; sensitivity 0.774; specificity 0.820). External validation confirmed its generalizability (AUC 0.720, 95% CI 0.670-0.771 and AUC 0.747, 95% CI 0.712-0.782). Decision curve analysis highlighted a substantial clinical net benefit. A user-friendly online prediction web page was also developed.

**Conclusions:**

We successfully developed and validated a dynamic, online nomogram for predicting ARHL in older adults. Comprising 6 readily available predictors, this model shows potential as a practical, online tool for proactive risk identification in primary care. However, further validation in larger and more diverse populations is essential to confirm its generalizability and real-world clinical utility.

## Introduction

Age-related hearing loss (ARHL) is the third most prevalent health condition among older adults, after hypertension and arthritis, and has become a significant public health issue [1]. The World Health Organization estimates that more than 1.5 billion people worldwide experience hearing loss, a number projected to reach 2.5 billion by 2050 [2]. In China, hearing loss affects 30% of adults older than 60 years and rises to 50% for those older than 70 years, totaling at least 70 million cases [3]. ARHL can hinder communication and social interaction, leading to greater risks of social isolation, depression, cognitive decline [4-6], frailty, and falls [7,8].

Despite these negative health consequences, routine hearing screening remains significantly underused, with rates below 25% in older populations [9,10]. Key barriers include limited patient awareness and financial constraints; for example, a diagnostic audiogram in China can cost ¥100 (US $14.1) to ¥300 (US $42.3), a substantial out-of-pocket expense for many older adults; these challenges are further compounded by limited time, training, and equipment among primary care clinicians [11-14]. Furthermore, high false-positive rates from some hearing screening methods can increase costs, prompting policymakers to adopt a cautious approach [3,15].

Leveraging primary health care centers for the management of hearing loss is the most suitable and cost-effective approach [16,17]. A preliminary prediction tool designed for these centers could accurately identify individuals with high risk for targeted audiometric screening, thereby optimizing referrals and specialist resources. While previous models have been developed using known risk factors for hearing impairment, such as age, environmental noise [18], comorbidities [19], exercise, and nutrition [20], their clinical applicability is often limited by small sample sizes, a lack of external validation, and reliance on complex variables.

Therefore, this study aimed to develop a streamlined nomogram-based prediction model for ARHL among community-dwelling older adults, using a large sample derived from health check-ups conducted in primary health care institutions in Beijing, China. Additionally, the model was externally validated in 2 independent datasets. An online tool was used to deploy the model as an interactive application, enabling primary health care providers to perform risk prediction without requiring any software installation or programming expertise. This study adhered to the Transparent Reporting of a Multivariable Prediction Model for Individual Prognosis or Diagnosis guidelines [21].

## Methods

### Study Population

**Figure 1:**
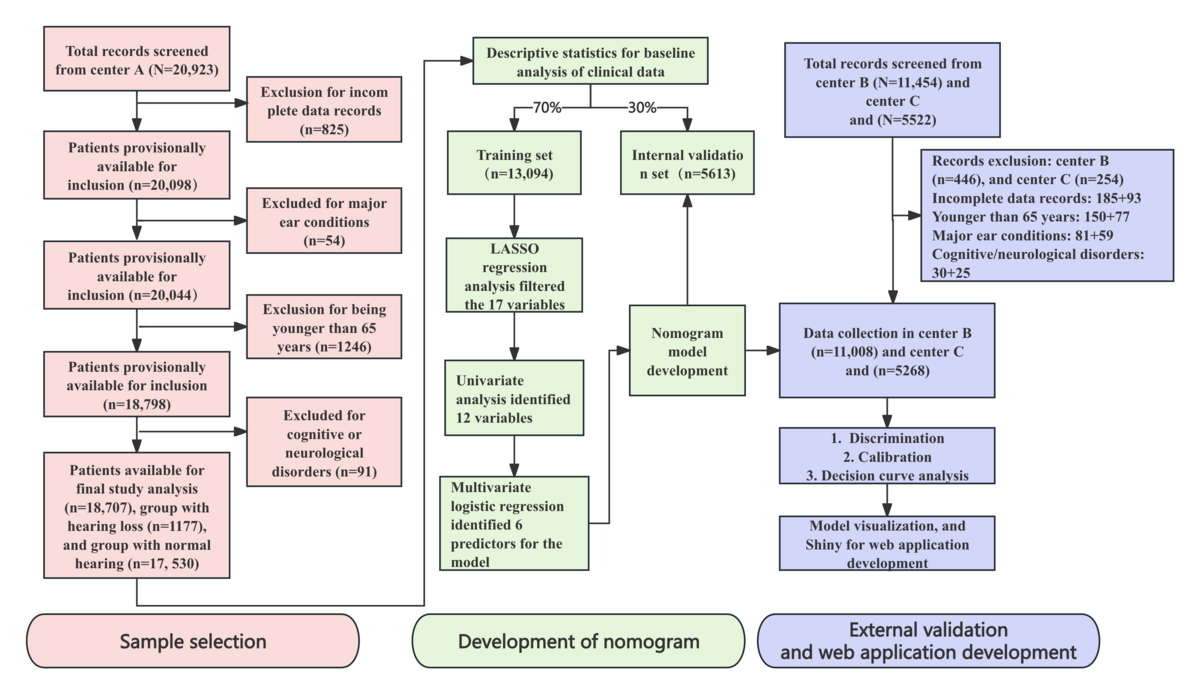
Flowchart for hearing loss prediction model development and validation.

This multicenter retrospective cohort study used electronic health examination records from 3 community primary health care centers (center A, center B, and center C) in Beijing, China. Data were collected from older adults who participated in the annual health checkup under the National Public Health Service Project between January 1, 2020, and October 30, 2023. The dataset included comprehensive information on demographics, medical history, physical examination results, and chronic disease records. For this study, the dataset from center A, which had the largest sample size, was used for model development and internal validation, while data from center B and center C were used for external validation. A detailed study design and sample selection are shown in Figure 1.

From center A, a total of 20,923 patient records were initially screened. First, 3.94% (825/20,923) records with incomplete data were excluded. After subsequently excluding 0.26% (54/20,923) individuals with major ear conditions, 5.96% (1246/20,923) individuals aged younger than 65 years, and 0.43% (91/20,923) individuals with cognitive or neurological disorders, a final cohort of 89.41% (18,707/20,923) participants was included in the analysis. The external validation cohorts were derived from center B and center C. Initially, 11,454 records from center B and 5522 records from center C were screened. After applying the exclusion criteria, a total of 3.89% (446/11,454) participants were excluded from center B and 4.60% (254/5522) from center C. This process resulted in final validation cohorts of 96.11% (11,008/11,454) participants from center B and 95.40% (5268/5522) from center C.

### Assessment of Hearing Loss

The outcome variable for this study was hearing loss, which was assessed using the whispered voice test as specified by the standardized protocol in the National Basic Public Health Service Standards (Third Edition) [22], issued by China’s National Health Commission. This screening was performed by trained primary health care professionals to ensure consistency across all centers.

Specifically, the whispered voice test, a widely recognized and validated screening tool, was used due to its simplicity, cost-effectiveness, and reliability in detecting hearing loss in clinical and community settings [23,24]. The protocol dictates the following procedure: the examiner stands approximately 0.5 m from the participant’s tested ear, positioned slightly behind them to prevent lip-reading. The participant’s nontested ear is occluded (blocked) by the examiner to isolate the tested ear. After a full exhalation, the examiner whispers a simple, standardized phrase (eg, What is your name?). Failure to correctly repeat the phrase was classified as the presence of hearing loss. For the purpose of our model, this binary outcome (presence vs absence of hearing loss) was used as the dependent variable [22].

### Sample Size Estimation

The determination of an adequate sample size for a multivariate predictive model requires a sufficient representation of individuals and outcome events relative to predictor parameters [25]. The combined sample size from 3 primary health care institutions provided robust support for using 33 predictive variables in both modeling and validation. Specifically, according to the widely applied 10 events per variable principle, at least 330 events (33 variables×10 events) are required [26]. Given an event rate of 0.063, this translates to approximately 5239 cases. The modeling dataset comprised 18,707 cases and 1177 events, which is more than 3 times the minimum requirement of 330 events. This large number of events per variable provides sufficient statistical power and minimizes the risk of model overfitting.

### Model Development and Validation

Variable selection was conducted using the least absolute shrinkage and selection operator (LASSO) regression, which applies a Least Absolute Deviations penalty to shrink some regression coefficients to 0, simplifying the model while preserving variables with strong predictive power. Tenfold cross-validation determined the optimal penalty parameter λ (λ_min), and variables with nonzero coefficients in the final model were chosen as candidate predictors [27,28]. Next, univariate analysis was performed on the LASSO-selected variables. Variables with *P* values <0.05 were considered for inclusion in the multivariate logistic regression model. A forward stepwise regression approach was applied to identify the final predictors to be included in the model. To handle missing data, a complete case analysis was used. This approach, which excludes records with any missing data on key variables, was considered appropriate because the proportion of such records was low across all cohorts (3.94% in center A, 1.62% in center B, and 1.68% in center C), consistently remaining below 5%.

Statistical analysis was performed using R software (version 4.2.2; R Foundation for Statistical Computing). To facilitate clinical application of the prediction model, we developed an interactive online application using the *Shiny* framework (RStudio, PBC). The final logistic regression model and coefficients were embedded into the server logic using the *rms* package to ensure consistency with the nomogram. A user-friendly interface was created with *Shiny*, *shiny Widgets*, and *shiny themes* packages, allowing real-time input and calculation of predicted risk. Dynamic visualizations of individualized risk were implemented using *plotly*, providing immediate graphical feedback. The application was deployed on a cloud-based *Shiny* server to allow online access without requiring local installation.

Model validation included 3 aspects: discrimination, calibration, and clinical utility [29]. Discrimination was assessed using the area under the receiver operating characteristic curve (AUC), sensitivity, specificity, and Matthews correlation coefficient to evaluate the model’s ability to distinguish between individuals with hearing loss and those without hearing loss. The calibration of the model was assessed using calibration plots generated through 1000 bootstrap resamples, in addition to the Brier score and mean absolute error, to evaluate the consistency between the predicted and actual observed hearing loss outcomes. Risk stratification strategy was based on the optimal cutoff value (the hearing loss risk probability when the Youden index is maximum, Youden=sensitivity+specificity−1) [30]. Finally, the clinical utility of the final model was evaluated using decision curve analysis.

### Ethical Considerations

The study was approved by the ethics committee of Capital Medical University, Beijing, China (Z2024SY071). Informed consent was obtained from participants during routine health examinations, and no additional consent was required for this secondary analysis. All data were anonymized and securely stored with access restricted to the research team. No financial or other compensation was provided, as the data were derived from routine public health services.

## Results

### Population Characteristics

A total of 18,707 participants were included in the analysis in center A. Of these, 44.20% (8268/18,707) were male and 55.80% (10,439/18,707) were female, with a mean age of 72.89 (SD 7.15, range 65-99) years. A total of 6.29% (1177/18,707) participants were identified as having hearing loss, while 93.71% (17,530 /18,707) had normal hearing. The participants were then randomly divided into training (13,094/18,707, 70%) and internal validation sets (5613/18,707, 30%) in a 7:3 ratio. Additionally, 96.11% (11,008/11,454) and 95.40% (5268/5522) of participants were included from center B and center C, respectively, for external validation.

### Model Development and Performance

**Figure 2:**
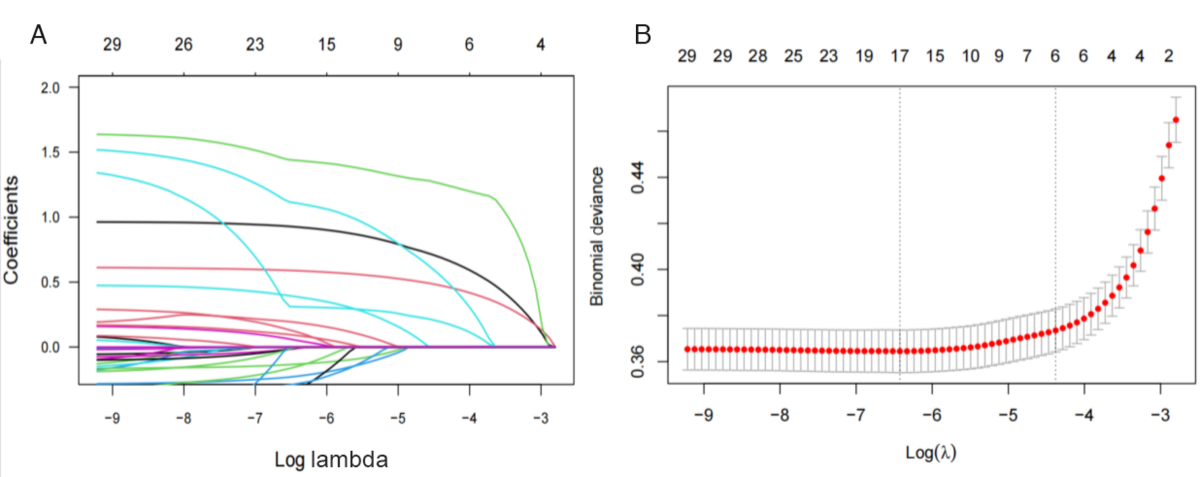
Feature selection using the least absolute shrinkage and selection operator (LASSO) regression. (A) The LASSO coefficient profile plot. Each colored line represents the coefficient path for 1 of the 33 candidate predictor variables as the regularization penalty (log lambda) increases. As the penalty grows, the coefficients of less important variables are shrunk toward 0. (B) Identification of the optimal penalization coefficient (λ) was achieved by 10-fold cross-validation and the minimum criterion. The left vertical line represents the mean square error, and the right vertical line represents the cross-validated mean square error within 1 SE of the minimum.

The most significant features selected by LASSO from the training set were used to identify potential predictors for the model. The screening process is detailed in Figure 2. Initially, 33 variables were identified, guided by systematic reviews, meta-analyses, and expert clinical opinions. These factors encompass demographic characteristics, lifestyle factors, physiological measurements, and chronic diseases. The model’s bias showed minimal variation within the interval (λ_1se_, λ_min_). When λ_min_ was set to 0.002 in the LASSO model, 17 variables exhibited nonzero coefficients, as shown in Table 1.

**Table 1:** Univariate analysis of potential predictors for hearing loss in the training set (n=13,094).

Variables		No hearing loss, n (%)	Hearing loss, n (%)	Chi-square *(*df*)*		*P* value
**Sex**					0.5 (1)	.49
	Male	6832 (52.2)	453 (3.5)			
	Female	5448 (41.6)	361 (2.8)			
**Age (years)**					677.3 (2)	<.001
	65-70	6467 (49.4)	152 (1.2)			
	71-80	3785 (28.9)	242 (1.8)			
	≥81	2028 (15.5)	420 (3.2)			
**Education level**					33.3 (2)	<.001
	Associate degree or above	2815 (21.5)	207 (1.6)			
	Middle or high school	6395 (48.8)	343 (2.6)			
	Primary school or below	3070 (23.4)	264 (2)			
**Marital status**					1.8 (1)	.19
	Married	9470 (72.3)	611 (4.7)			
	Other (Never married, divorced, or separated, widowed)	2810 (21.5)	203 (1.6)			
**Waist circumference**					0.3 (1)	.56
	Normal	7737 (59.1)	504 (3.8)			
	Abnormal	4543 (34.7)	310 (2.4)			
**BMI (kg/m^2^)**				8.0 (2)		.02
	18.5-29.9	11,708 (89.4)	759 (5.8)			
	＜18.5	181 (1.4)	15 (0.1)			
	＞30.0	391 (3)	40 (0.3)			
**Physical function**					483.1 (1)	<.001
	Able to perform smoothly	12,203 (93.2)	739 (5.6)			
	Unable to perform any task independently	75 (0.6)	77 (0.6)			
**Exercise frequency**					886.0 (3)	<.001
	Everyday	7592 (58)	205 (1.6)			
	More than once a week	1826 (13.9)	79 (0.6)			
	Occasionally	970 (7.4)	75 (0.6)			
	Not exercising	1892 (14.4)	455 (3.5)			
**Dietary habits**					57.2 (3)	<.001
	Balanced diets	11,411 (87.1)	703 (5.4)			
	Primarily vegetarian diet	527 (4)	59 (0.5)			
	Primarily meat-based diet	182 (1.4)	21 (0.2)			
	Preference for sugar, salt, and oil	160 (1.2)	31 (0.2)			
**Smoking**					11.9 (2)	.003
	Never smoked	11,107 (84.8)	760 (5.8)			
	Former smoker	360 (2.7)	25 (0.2)			
	Current smoker	813 (6.2)	29 (0.2)			
**Alcohol consumption frequency**					13.5 (2)	.001
	Never	11,138 (85.1)	768 (5.9)			
	Occasionally	486 (3.7)	15 (0.1)			
	Frequently or daily	656 (5)	31 (0.2)			
**Hypertension**					19.7 (1)	<.001
	No	4785 (36.5)	253 (1.9)			
	Yes	7495 (57.2)	561 (4.3)			
**Chronic obstructive pulmonary disease**					0.01 (1)	.90
	No	12,115 (92.5)	804 (6.1)			
	Yes	165 (1.3)	10 (0.1)			
**Diabetes**					0.8 (1)	.37
	No	3730 (28.5)	260 (2)			
	Yes	8550 (65.3)	554 (4.2)			
**Stroke**					2.1 (1)	.15
	No	10,365 (79.2)	671 (5.1)			
	Yes	1915 (14.6)	143 (1.1)			
**Left systolic blood pressure (mm Hg)**					5.4 (1)	.02
	90-140	10,573 (80.7)	725 (5.5)			
	＜90 or ＞140	1707 (13)	89 (0.7)			
**Right systolic blood pressure (mm Hg)**					5.7 (1)	.02
	90-140	10,597 (80.9)	727 (5.6)			
	＜90 or ＞140	1683 (12.9)	87 (0.7)			

First, univariate analysis was conducted on the 17 variables identified through LASSO regression to evaluate their independent associations with hearing loss. The detailed results of this analysis, including odds ratios and *P* values for all variables, are presented in Table 1. The results indicated that age, education level, marital status, BMI, physical function, exercise frequency, dietary habits, smoking, alcohol consumption frequency, hypertension, left systolic blood pressure, and right systolic blood pressure were significantly associated with hearing loss.

**Figure 3:**
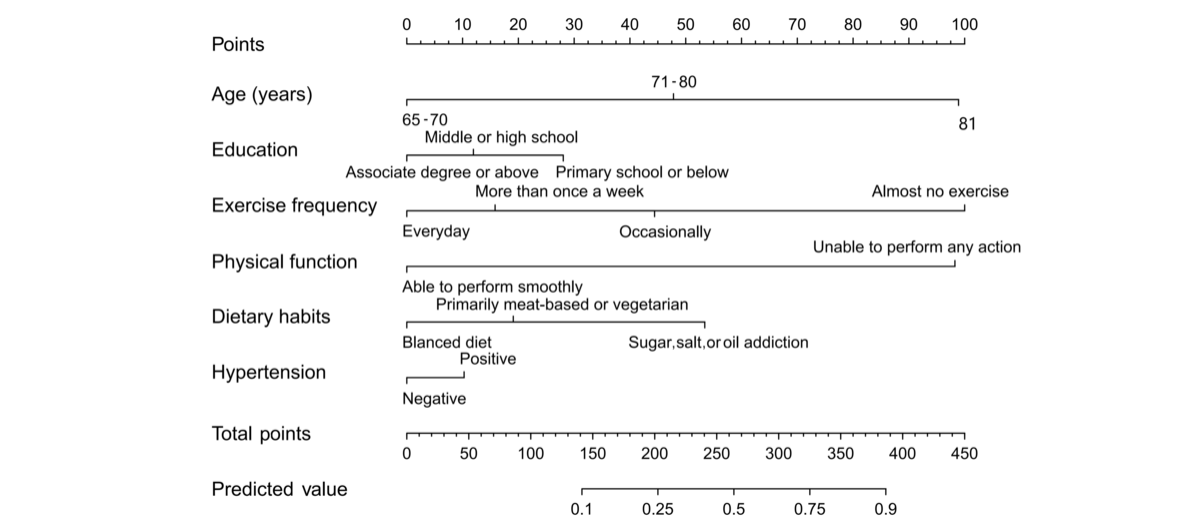
Nomogram to predict the probability of hearing loss in community-dwelling older adults.

Subsequently, a forward stepwise regression approach selected 6 predictors—age, education, exercise frequency, physical function, dietary habits, and hypertension—which were then included in the multivariate logistic regression model (Table 2). A user-friendly nomogram was developed, as illustrated in Figure 3. The optimal cutoff value was 0.805 based on the maximum Youden index. Accordingly, the population was classified into low (predicted probability <80.5%) and high (predicted probability≥80.5%) hearing loss risk groups. A comparison of the model’s predicted risk classifications against the observed outcomes is presented in Table 3. For example, in the training set, of the 814 individuals, the model correctly identified 639 (78.5%) with hearing loss (true positives) and of the 12,280 individuals, 10,099 (82.2%) with normal hearing (true negatives), corresponding to the sensitivity and specificity values reported.

In the training set, the model achieved an AUC of 0.828 (95% CI 0.813-0.842), sensitivity was 0.785 (639 out of 814 participants who actually developed hearing loss were correctly predicted as high-risk), specificity was 0.822 (10,099 of the 12,280 participants who did not develop hearing loss were correctly predicted as low-risk), and balanced accuracy was 0.804. In the internal validation set, AUC was 0.806 (95% CI 0.782-0.831), sensitivity was 0.774, specificity was 0.820, balanced accuracy was 0.797, and Matthews correlation coefficient was 0.350.

**Table 2:** Results of the multivariate logistic regression model for predicting hearing loss.

Predictor		β (SE)	OR^a^ (95% CI)	*P* value
Intercept		−4.46 (0.131)	—^b^	<.001
**Age (years)**				
	65-70 (reference)	—	—	—
	71-80	1.15(0.108)	3.16 (2.56-3.91)	<.001
	≥81	1.97 (0.108)	7.19 (5.81-8.89)	<.001
**Education level**				
	Primary school or below (reference)	—	—	—
	Middle or high school	−0.34 (0.095)	0.71 (0.59-0.86)	<.001
	Associate degree or above	−0.49 (0.107)	0.61 (0.50-0.76)	<.001
**Exercise frequency**				
	Every day (reference)	—	—	—
	More than once a week	0.39 (0.133)	1.48 (1.14-1.91)	.003
	Occasionally	0.75 (0.144)	2.13 (1.61-2.81)	<.001
	Almost no exercise	1.84 (0.091)	6.3 (5.27-7.52)	<.001
**Physical function**				
	Able to perform smoothly (reference)	—	—	—
	Unable to perform any action	1.87 (0.176)	6.47 (4.58-9.14)	<.001
**Dietary habits**				
	Balanced diets (reference)	—	—	—
	Primarily meat-based or vegetarian	0.37 (0.135)	1.45 (1.11-1.89)	.006
	Sugar, salt, and oil addiction	0.89 (0.23)	2.43 (1.55-3.82)	<.001
**Hypertension**				
	Negative (reference)	—	—	—
	Positive	0.26 (0.083)	1.29 (1.09-1.52)	.002

^a^OR: odds ratio.

^b^Not available.

**Table 3:** Comparison of model-predicted risk stratification and observed hearing loss across training and validation cohorts.

Predicted risk group		Observed hearing loss, n	Observed no hearing loss, n	Row total, n
**Training set**				
	Model-predicted high risk^a^	639	2181	2820
	Model-predicted low risk^b^	175	10,099	10,274
	Column total	814	12,280	13,094
**Internal validation set**				
	Model-predicted high risk^a^	281	946	1227
	Model-predicted low risk^b^	82	4304	4386
	Column total	363	5350	5613
**External validation set** **A**				
	Model-predicted high risk^a^	87	1285	1372
	Model-predicted low risk^b^	39	9597	9636
	Column total	126	10,882	11,008
**External validation set** **B**				
	Model-predicted high risk^a^	148	791	939
	Model-predicted low risk^b^	89	4240	4329
	Column total	237	5031	5268

^a^The model-predicted low-risk group was defined as predicted probability <80.5%.

^b^The model-predicted high-risk group was defined as predicted probability ≥80.5%. Percentages are omitted because row percentages and column percentages represent different concepts. Absolute counts and all marginal totals are provided to allow direct calculation of any desired proportion.

### External Validation

**Figure 4:**
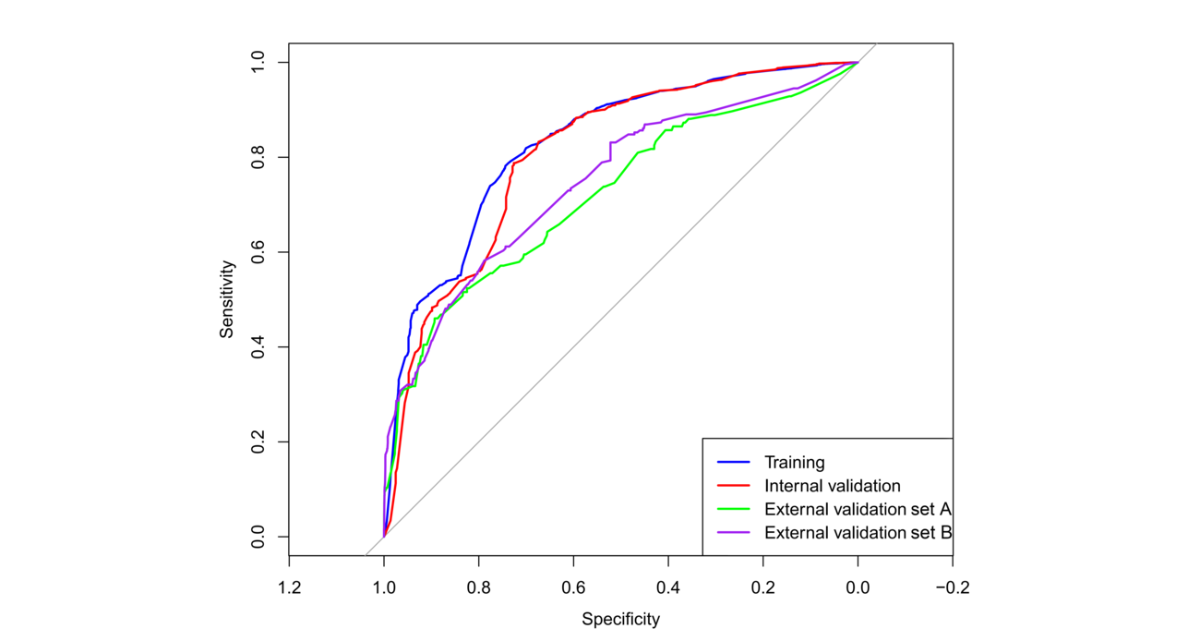
Receiver operating characteristic curves of the prediction model for hearing loss in community-dwelling older adults in the training and validation sets.

In external validation set A, the AUC was 0.720 (95% CI 0.670-0.771), with a sensitivity of 0.690, a specificity of 0.882, a balanced accuracy of 0.786, and a Matthews correlation coefficient of 0.204. In external validation set B, the model achieved an AUC of 0.747 (95% CI 0.712-0.782), with a sensitivity of 0.624, a specificity of 0.843, a balanced accuracy of 0.734, and a Matthews correlation coefficient of 0.270. These results highlight the model’s good predictive performance for identifying hearing loss in older adults. The receiver operating characteristic curves are shown in Figure 4.

**Figure 5:**
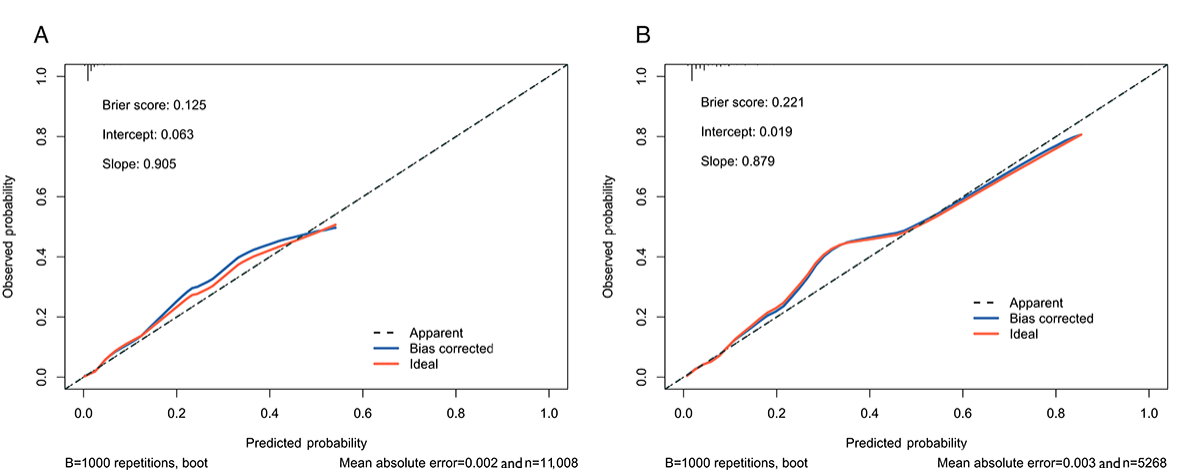
Calibration plots of the prediction model for hearing loss in community-dwelling older adults in external validations sets. (A) Calibration plot for external validation set A. (B) Calibration plot for external validation set B.

The nomogram’s calibration plots are shown in Figure 5. Quantitative metrics supported the visual assessment of calibration. In the external validation set A, the Brier score was 0.125 and the mean absolute error was 0.002. In the external validation set B, these values were 0.221 and 0.003, respectively. Lower Brier scores and mean absolute error values suggest that the model provides probability estimates close to the true risk, helping clinicians avoid unnecessary interventions for patients with low risk and missed diagnoses in patients participants with high risk.

**Figure 6:**
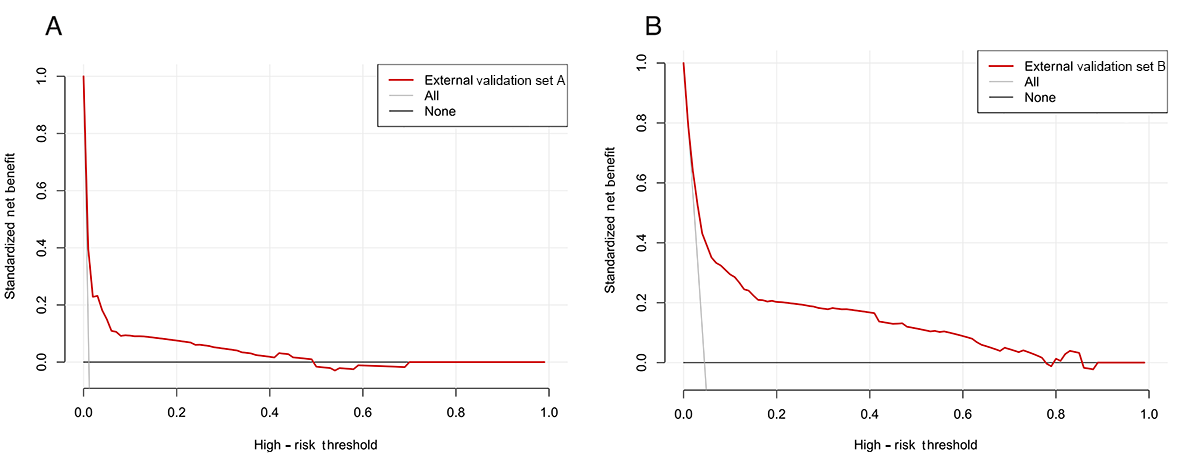
Decision curve analysis of the prediction model for hearing loss in community-dwelling older adults in external validation sets. (A) Decision curve analysis for external validation set A. (B) Decision curve analysis for external validation set B.

Figure 6 presents the decision curves for the nomogram. The high-risk threshold probability represents the predicted probability of hearing loss, above which clinicians would consider routine audiometric screening and early interventions, such as lifestyle modifications. In the decision curve analysis plots for both cohorts, the model (red line) consistently demonstrated greater net benefit than the default strategies (treat all or none) across a conservative overlapping threshold range of approximately 5% to 75%. Using the model to guide ARHL prediction and implement routine screening and early interventions provided an additional net benefit of up to 20% compared with alternative strategies that assume all patients are either severely or nonseverely affected.

### Visualization of Predictive Models: Development of a User-Friendly Web Page

**Figure 7:**
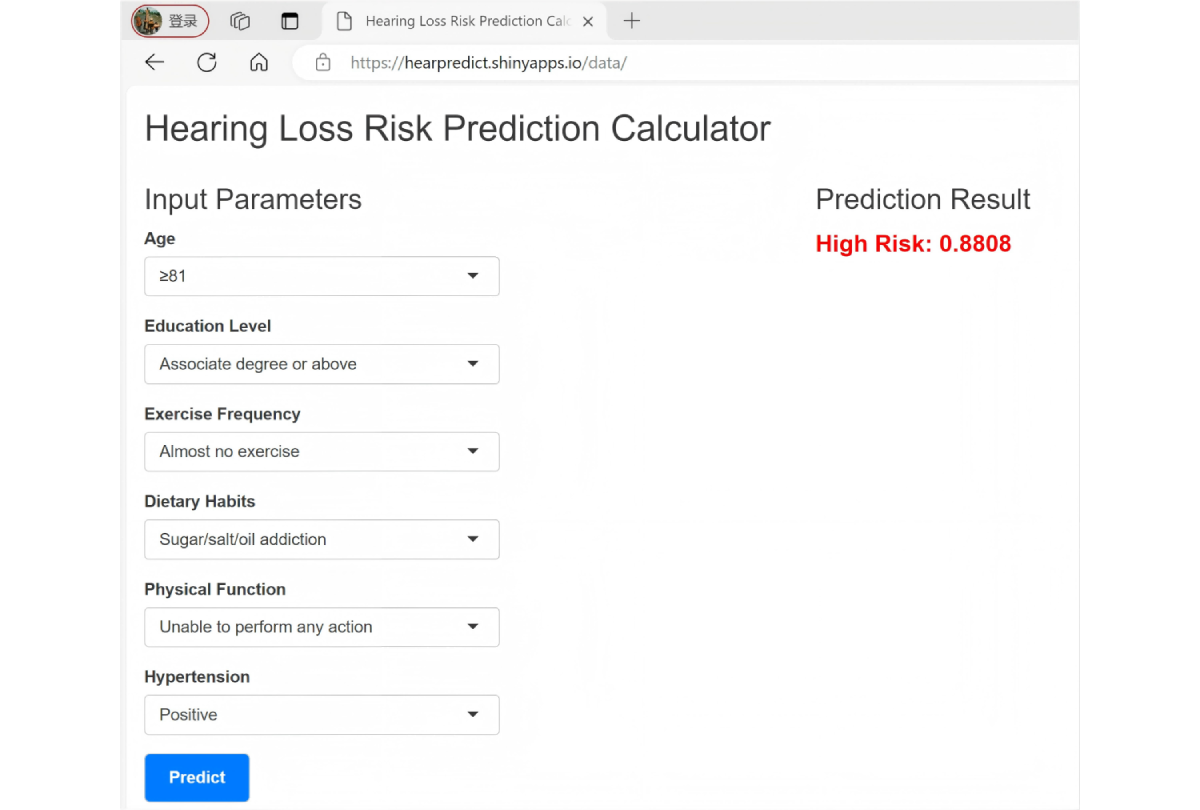
Web page–based estimation of hearing loss likelihood in community-dwelling older adults.

To facilitate the clinical application of our nomogram, we developed an interactive and user-friendly web application using the R Shiny framework. The application features a simple, single-panel user interface where clinicians can input a patient’s 6 predictor variables—age, education, exercise frequency, physical function, dietary habits, and hypertension—using a combination of numeric input fields and dropdown menus (Figure 7).

Upon clicking a *Predict* button, the tool instantly computes and displays the patient’s predicted probability of hearing loss. The output is presented as a clear percentage, along with a corresponding risk stratification (*High risk* or *Low risk* based on the 80.5% cutoff) to aid in immediate clinical decision-making. The web application is publicly accessible and requires no software installation or programming knowledge, ensuring its broad utility for primary care providers [31].

## Discussion

### Principal Findings

The principal finding of this large, multicenter study is the development and external validation of a practical, 6-predictor nomogram for predicting hearing loss in community-dwelling older adults. Our model, which uses readily available data from routine health checkups (age, education, exercise frequency, physical function, dietary habits, and hypertension), demonstrated good discriminative ability (AUC>0.720 in all cohorts) and calibration.

A key clinical implication of this finding is the model’s potential to shift primary care from reactive secondary screening to proactive primary prevention. The online tool, developed as a proof of concept, illustrates how this model could be integrated into clinical workflows. For example, if embedded within electronic medical record systems, it could provide real-time risk scores, creating a risk-stratified screening pathway. This would allow general practitioners to prioritize formal audiometry and targeted interventions for individuals at the highest risk, potentially improving the efficiency of health care resource allocation.

### Comparison With Prior Work

Our risk prediction model incorporates demographic, lifestyle, and chronic disease factors, aligning broadly with previous studies. It is well established that age is strongly associated with hearing loss. Hearing decline along with aging is primarily attributed to progressive degeneration of cochlear sensory hair cells, stria vascularis, and spiral ganglion neurons, leading to impaired sound transmission [18,32]. Consistent with previous studies, we found a higher risk of hearing loss in individuals with lower education levels [33,34]. This may be attributed to its correlation with lower socioeconomic status [35], which can limit health care access and use, ultimately leading to poorer health status. Additionally, lower education levels are often associated with unhealthy lifestyle attributes (eg, smoking, alcohol consumption, and exercise habits) that were included in our model [36]. Interestingly, while recent studies have suggested a potential link between diabetes and hearing loss [37,38], our study did not identify a statistically significant association between the two. Instead, hypertension was included as a predictor in the model. It may contribute to hearing decline by reducing blood supply to the stria vascularis, a cochlear structure dependent on terminal arteries without collateral circulation [39,40].

Recent studies have focused on a prediction model for hearing loss. Ge et al [41] developed a logistic regression model for ARHL using 18 key factors based on 401 older adults. Their model achieved an AUC value of 0.777, and the sensitivity and specificity were 70.9% and 75.3%, respectively; however, due to limited sample size and absence of internal and external validation, further scrutiny is required regarding its predictive performance. Similarly, Wang et al [42] developed a predictive model comprising 22 features. While the model achieved an AUC of 0.864 and 0.844 during training and internal validation, the large number of variables and complex operation may hinder its clinical applicability and generalizability. Predictive models should strike a balance between performance and practicality, aiming to achieve the highest possible predictive accuracy with the fewest possible variables [43].

### Strengths and Limitations

The primary strengths of this study include its large, multicenter sample, which enhances the robustness and generalizability of our findings, and a rigorous variable selection process. The resulting 6-predictor model is simple, and its variables are accessible from routine physical examinations without requiring specialized tests. Furthermore, the development of an online tool highlights its potential feasibility for real-world implementation. Finally, the selection of an interpretable *white-box* model (logistic regression) was a deliberate choice. While more complex *black-box* algorithms may offer marginal gains in accuracy, their lack of transparency is a significant barrier to clinical adoption. Our model achieves an optimal balance between its robust, externally validated performance and the essential need for interpretability, making it well suited for guiding real-world clinical decisions.

However, this study is not devoid of limitations. First, our study was conducted exclusively in primary health care centers within Beijing. While we attempted to mitigate this by validating across 3 distinct centers, the shared urban setting may influence the results by limiting the model’s generalizability. Second, our model intentionally omits key risk factors such as genetic predisposition, long-term noise exposure, and ototoxic medication use. This was a necessary trade-off to create a practical tool based solely on variables routinely collected in health checkups. However, we acknowledge that the absence of these predictors may limit the model’s accuracy in populations where such factors are highly prevalent. Third, hearing loss was assessed using the whispered voice test rather than pure-tone audiometry. To mitigate this, all professionals followed a standardized protocol. However, this method may influence the results by underestimating mild hearing loss and being subject to interexaminer variability. Fourth, several predictors (eg, physical function, exercise frequency, and dietary habits) were based on self-report, which may be prone to information bias and reduce predictive precision. Finally, as a retrospective cohort study, our analysis can suggest temporal relationships between exposures and outcomes but cannot establish definitive causal inferences due to potential confounding and selection biases.

### Future Directions and Conclusions

Building on this work, several avenues for future research are recommended. The immediate priority is to validate the current nomogram in broader and more diverse geographic and demographic cohorts, using pure-tone audiometry as the gold standard outcome for a more precise assessment. Future prospective studies could also enhance the model’s accuracy by incorporating key variables not available in our dataset, such as genetic predisposition, long-term noise exposure, and objective measures of physical activity. Finally, as the current online tool is a proof of concept, dedicated implementation research is required. This should address the practical challenges of integrating the tool into diverse electronic medical record systems and evaluate its real-world impact on clinical decision-making, patient outcomes, and cost-effectiveness.

In conclusion, this study provides a validated, practical tool that shows promise for identifying older adults at high risk of hearing loss. If confirmed in future studies, this nomogram could become a valuable asset in the primary prevention of ARHL.

## Abbreviations

ARHL: age-related hearing loss
AUC: area under the receiver operating characteristic curve
LASSO: least absolute shrinkage and selection operator

## Data Availability

The datasets generated or analyzed during this study are not publicly available due to patient privacy regulation but are available from the corresponding author on reasonable request.
